# Satisfaction-Related Well-Being Among Working Young Adults and Their Parents: Core Self-Evaluations and Life Values in a Preregistered Cross-Sectional Study

**DOI:** 10.3390/brainsci16070761

**Published:** 2026-07-20

**Authors:** Radosław B. Walczak, Raluca Sassu, Hadar Nesher Shoshan, Maria Latusek-Mierzwa

**Affiliations:** 1 Institute of Psychology, University of Opole, 45-052 Opole, Poland; 2 Human Behavior and Development Research Laboratory, Lucian Blaga University of Sibiu, 550064 Sibiu, Romania; raluca.sassu@ulbsibiu.ro; 3 Department of Psychology/Work and Organizational Psychology, Johannes Gutenberg University Mainz, 55122 Mainz, Germany

**Keywords:** Generation Z, parent–child dyads, work satisfaction, life satisfaction, core self-evaluations, Schwartz values, financial situation, young adults

## Abstract

Background: Previous research has documented generational differences in life satisfaction and work-related attitudes, but less is known about how work and life satisfaction differ within parent–child dyads. This preregistered study examined work satisfaction, life satisfaction, core self-evaluations, personal values, and financial situation among working young adults and their working parents in Poland. Methods: The study used a preregistered, cross-sectional, observational parent–child dyadic design. The final analytic sample consisted of 87 complete dyads, corresponding to 174 individual participants. Parent–child responses were linked using anonymised self-generated codes. Group differences in work satisfaction, life satisfaction, and core self-evaluations were tested using paired Yuen’s trimmed *t*-tests. Preregistered predictors of work satisfaction and life satisfaction were tested using linear mixed-effects models with dyads as a random intercept. Results: Parents reported higher work satisfaction and higher core self-evaluations than their young-adult children, whereas the expected group difference in life satisfaction was not supported. Core self-evaluations positively predicted work satisfaction, whereas the preregistered financial situation composite and achievement value did not. A supplementary component-level analysis showed heterogeneous associations among the individual financial indicators. Work satisfaction was positively associated with life satisfaction, whereas the preregistered Schwartz values were not significantly associated with life satisfaction. Conclusions: The findings suggest that parent–child differences in satisfaction-related outcomes are more visible in work satisfaction and core self-evaluations than in global life satisfaction. Core self-evaluations emerged as the most consistent psychological predictor of work satisfaction. The value-based hypotheses were not supported, although these null findings warrant caution because several brief PVQ scales had low reliability.

## 1. Introduction

### 1.1. Satisfaction-Related Well-Being in Young Adults Entering Work

The transition from late adolescence into young adulthood increasingly involves early participation in paid work, often alongside higher education. For many young adults, work is not yet a stable full-time career role but a context of part-time, temporary, or student employment. This makes work a relevant setting for understanding satisfaction-related well-being during a developmental period in which individuals are forming expectations about autonomy, financial independence, future security, and their place in the labour market.

The present study focuses on two related but distinct indicators of satisfaction-related well-being: work satisfaction and life satisfaction. Work satisfaction reflects how individuals evaluate their work, whereas life satisfaction refers to a broader cognitive evaluation of life as a whole. These constructs are not equivalent to clinical mental health or mental illness, but they are commonly treated as important indicators of subjective well-being and psychological functioning. In the context of young adults entering employment, work satisfaction may be particularly informative because paid work can shape perceived competence, agency, economic independence, and future life planning.

Previous research provides a broad background for expecting differences in well-being and work-related evaluations between younger and older cohorts. Grelle et al. [1] reported that Generation Z showed poorer mental health indicators than Generation X during the initial COVID-19 pandemic, including higher rates of provisional major depressive disorder and generalized anxiety disorder, as well as higher perceived stress, loneliness, and fatigue. Krokstad et al. [2] documented divergent age-related trends in Norway, showing increasing mental distress among adolescents and young adults while depressive symptoms declined among older adults. Moreno-Agostino et al. [3] similarly found that younger generations in the United Kingdom reported poorer mental and social well-being during the first year of the COVID-19 pandemic, with widening inequalities in anxiety over time. Borg et al. [4] further showed that contemporary adolescent cohorts reported higher and increasing mental health problems compared with earlier cohorts.

These findings provide a broader context for examining whether satisfaction-related well-being differs between working young adults and older family members. In the present study, the younger participants are treated descriptively as Generation Z, following Dimock’s [5] cohort boundary, whereas their parents mostly fall within the Generation X age range. However, the study is not designed to isolate pure cohort effects. Because the design compares working young adults with their working parents, generation is necessarily confounded with age, life stage, family role, labour-market experience, and accumulated resources. Therefore, generational labels are used descriptively, and the findings are interpreted primarily as parent–child and life-stage differences within matched family dyads.

Research on work-related attitudes also indicates that generational differences in job satisfaction tend to be modest, yet some evidence is consistent with the preregistered expectation that Generation X workers report higher work satisfaction than Generation Z workers. Ravid et al. [6], in a meta-analysis of generational differences at work, found only small differences in job satisfaction across generational cohorts and emphasized that such differences may partly reflect age, tenure, and work experience rather than generation alone. Jung and Shin [7] compared young adults of the same age across historical periods and found that Millennials and Generation Z reported lower job satisfaction and organizational commitment than Generation X young adults, suggesting that work attitudes may also be shaped by broader socioeconomic and labour-market conditions. Nichols and Smith [8] reported that Generation Z workers showed lower job satisfaction than older cohorts and placed greater emphasis on progress, challenge, mental health, and employer commitments to diversity. This led us to formulate the preregistered hypothesis H1: Gen X workers will report higher work satisfaction than Gen Z workers. At the same time, the literature suggests that such differences should be interpreted with caution and in relation to broader contextual factors, including career stage, work experience, and labour-market conditions, rather than being attributed solely to generational labels.

On this basis, the present study examines whether working young adults report lower work satisfaction and life satisfaction than their working parents. Because the preregistered hypotheses used generational labels, these labels are retained in the hypothesis statements, but the interpretation of the findings is centered on parent–child and life-stage comparison rather than on pure generational explanation.

### 1.2. Parent–Child Work Values and the Dyadic Context

Parent–child dyadic research offers a useful framework for studying work-related attitudes because parents and their adult children are linked through shared family context while occupying different life stages and work biographies. In the present study, the term dyadic refers to the matched parent–child data structure: each young adult was linked to one working parent, and the analyses accounted for non-independence within dyads. This approach assumes that family-linked comparisons provide a meaningful context for examining similarities and differences in satisfaction-related outcomes, as shared background may shape attitudes while differing life stages introduce variation. At the same time, this assumption implies a limitation: observed associations reflect both shared family context and individual life-stage factors, which cannot be disentangled within the present design.

Prior research nevertheless suggests that family context matters for work-related orientations. Cemalcilar et al. [9], in a meta-analysis of intergenerational transmission of work values, found significant but moderate parent–child similarity in work values, indicating that parents may contribute to children’s work-related orientations while leaving substantial room for other socialization influences. Kraaykamp et al. [10] similarly argued that work attitudes and values are shaped by multiple environments, including family, education, and work experience. Sümer et al. [11] further showed that parent–child similarity in work values may depend on family climate and parenting behaviours, although such effects are not uniform across countries and family contexts.

Research on work-related socialization also links parental work experiences with children’s developing work beliefs. Steiner et al. [12] found that children’s perceptions of parental job satisfaction were associated with children’s work centrality, suggesting that parental work experiences may become meaningful for children’s understanding of work. French et al. [13] extended dyadic work–family research by showing that stress processes may be linked across parents and adolescents. This body of evidence supports the relevance of studying work-related attitudes within family-linked data, but it has focused mainly on work values, work centrality, and strain. Less is known about how work satisfaction and life satisfaction differ between working young adults and their working parents within matched dyads.

The present study addresses this gap by examining satisfaction-related outcomes in Polish parent–child dyads. Its dyadic contribution lies in the matched comparison of young adults and their parents within the same family context. This design allows for a more context-sensitive comparison than studies based on unrelated age groups, while still requiring caution against interpreting the findings as evidence of intergenerational transmission or pure generational effects.

### 1.3. Work Satisfaction and Life Satisfaction

Work satisfaction and life satisfaction are theoretically related but distinct evaluative outcomes. Work satisfaction refers to an individual’s evaluation of their work, whereas life satisfaction reflects a broader assessment of life as a whole. Work satisfaction has a central place in organizational psychology because it is associated with employee well-being and with organizationally relevant outcomes such as performance, absenteeism, turnover intentions, organizational commitment, and occupational health. Filipkowski and Derbis [14], Kim et al. [15], Spector [16], and Steel et al. [17] all point to the importance of job or work satisfaction as both an individual and organizational outcome. Life satisfaction, by contrast, integrates evaluations across multiple domains, including work, family, leisure, and broader living conditions, and is usually treated as a cognitive component of subjective well-being. Diener et al. [18], Sirgy [19], Unanue et al. [20], and Kim et al. [15] all locate life satisfaction within this broader well-being framework.

The link between work satisfaction and life satisfaction has been explained through spillover, bottom-up, and top-down perspectives. The spillover perspective proposes that experiences in work and non-work life mutually transfer across domains, producing a positive association between work satisfaction and life satisfaction. Unanue et al. [20] describe this relationship as part of a broader work–life satisfaction process. Bottom-up accounts treat work satisfaction as one domain-specific contributor to overall life satisfaction, whereas top-down accounts emphasize that broader life satisfaction and stable dispositions may also influence how people evaluate work. Steel et al. [17] and Bialowolski and Weziak-Bialowolska [21] show that personality and life satisfaction are important for understanding job satisfaction and provide longitudinal evidence for reciprocal effects between job satisfaction and life satisfaction. Consistent with these perspectives, we hypothesize that work satisfaction will be positively related to life satisfaction (H6).

This relationship may be especially relevant in parent–child comparisons because working young adults and their parents differ in work biography, financial responsibilities, and family roles. For young adults, work may be closely tied to the transition toward independence. For parents, work may be embedded in a longer employment history and more stable economic position. Therefore, the present study examines both mean-level parent–child differences in satisfaction and the association between work satisfaction and life satisfaction within the dyadic data structure.

### 1.4. Core Self-Evaluations and Satisfaction

Core self-evaluations (CSEs) refer to a broad dispositional appraisal of one’s own worth, competence, control, and emotional stability, integrating self-esteem, generalized self-efficacy, locus of control, and emotional stability [22,23]. This construct is relevant to satisfaction research because individuals with higher CSEs tend to evaluate both themselves and their circumstances more positively, which may shape how they experience and interpret their work. Prior studies by Judge et al. [24] show that CSE predicts job and life satisfaction directly and indirectly while later evidence [25] identifies CSE as one of the strongest dispositional predictors of work satisfaction. Longitudinal findings by Wu and Griffin [26] further suggest that CSE is associated not only with higher job satisfaction but also with subsequent growth in work satisfaction.

Evidence from different cultural contexts also supports the relevance of CSE for satisfaction outcomes. CSE has been linked with job satisfaction and life satisfaction in Japanese and European samples [27,28,29], and Polish evidence from the study by Walczak and Derbis [30] indicates that CSE predicts work satisfaction among Polish workers, even when assessed indirectly by an important other. These findings support the expectation that CSE will be a significant predictor of work satisfaction in the present sample (H2).

The broader literature on younger people’s well-being provides an additional, though indirect, rationale for expecting parent–child differences in CSE. Fínez Silva and Morán Astorga [31] found that CSE was positively related to positive functioning among adolescents and young adults, while Rey and Extremera [32] showed that CSE was negatively related to perceived stress and positively related to life satisfaction. Because emotional stability is one component of CSE, the higher levels of anxiety, stress, and distress reported among younger cohorts in prior research may be consistent with lower CSE in working young adults. This does not establish a generational mechanism, but it supports examining whether working young adults differ from their parents in CSE; accordingly, we hypothesize that Gen X parents will report higher levels of CSE than Gen Z young adults (H3).

### 1.5. Financial Situation as a Contextual Correlate of Work Satisfaction

Financial situation represents a contextual condition that may shape how individuals evaluate their work. Work provides not only meaning, identity, and social participation, but also income, material security, and the possibility of planning future life arrangements. When employment is perceived as supporting financial adequacy and control over one’s living conditions, it may contribute to more favourable evaluations of work.

This argument is relevant across both members of the dyad, but it may be especially salient for young adults entering work. At this life stage, paid work often coincides with the transition from economic dependence to economic independence. Young adults usually have limited accumulated capital and may still live with their parents or have recently moved into rented accommodation. Their work satisfaction may therefore depend partly on whether employment makes independent living and future housing security seem attainable. Recent evidence supports treating financial situation as more than objective income alone. Acton et al. [33] showed that perceived income adequacy may capture socioeconomic position differently from household income, particularly across different social and household contexts. Knies [34] demonstrated that income and material deprivation are relevant to young people’s life satisfaction, especially as young people become more aware of financial constraints.

In the present study, financial situation is therefore treated as a contextual correlate of work satisfaction, with particular relevance for young adults’ transition into economic independence. In line with the preregistered plan, financial situation was operationalized as a composite of two relative-income indicators and housing situation, reflecting perceived economic adequacy and housing-related security. We hypothesize that financial situation will predict work satisfaction (H5).

### 1.6. Personal Values and Satisfaction

Personal values provide a theoretical basis for linking life priorities with satisfaction outcomes. In Schwartz’s approach [35,36], values are broad, trans-situational goals that guide evaluation, choice, and action across contexts. Values are organized as a motivational continuum: adjacent values express compatible goals, whereas opposing values express motivational tensions [36]. This framework is useful in the present study because life satisfaction may reflect not only external circumstances, but also the extent to which people can evaluate their lives as coherent with personally important goals.

The preregistered hypotheses focused on selected values from Schwartz’s framework. In line with H7, conformity, tradition, benevolence, self-direction, and hedonism will be positively related to life satisfaction. Conformity concerns restraint of actions that could violate social expectations or harm others and may therefore be linked with life satisfaction through social order and reduced interpersonal conflict. Tradition refers to respect, commitment, and acceptance of cultural, family, or religious customs, and may support life satisfaction by providing continuity, identity, and belonging. This argument is particularly relevant in Poland, where conservation values may be positively related to well-being when they fit the broader sociocultural context, as shown by Bojanowska and Urbańska [37]. Benevolence concerns preserving and enhancing the welfare of close others, which may contribute to life satisfaction through reliable close relationships and prosocial involvement. Self-direction refers to independent thought and action, and may support life satisfaction through autonomy, agency, and the ability to pursue one’s own goals. Hedonism concerns pleasure and enjoyment and may be linked with life satisfaction through the experience of positive affect and gratification, as suggested by Sagiv and Schwartz [38].

At the same time, value–well-being associations should not be treated as simple or guaranteed. Ostermann et al. [39] showed that the realization of personal values may be important for mental health and life satisfaction, suggesting that the ability to enact values may matter more than value importance alone. This distinction is relevant because the present study measured value priorities, not value realization. Therefore, the value hypotheses are treated as preregistered theoretical expectations, while their interpretation requires caution.

Achievement is examined separately as a predictor of work satisfaction. In Schwartz’s theory [35,36], achievement refers to personal success through the demonstration of competence according to social standards. This value is theoretically relevant to work satisfaction because paid work is a central setting in which competence, performance, recognition, and socially evaluated success can be expressed. Ismail et al. [40] also showed that personal values may be related to job performance and job satisfaction across job categories. Accordingly, we hypothesize that achievement will be positively related to work satisfaction (H8).

### 1.7. The Present Study

The present preregistered study examines satisfaction-related well-being among working young adults and their working parents in Poland. The study combines three elements. First, it compares young adults and parents in work satisfaction, life satisfaction, and core self-evaluations. Second, it examines whether work satisfaction is associated with life satisfaction. Third, it tests whether selected individual, contextual, and motivational variables—core self-evaluations, financial situation, and personal values—are associated with satisfaction outcomes.

The study uses a matched parent–child design. This design allows comparisons between family-linked young adults and parents and permits statistical control of non-independence within dyads. However, it does not isolate generation from age, life stage, family role, work experience, or accumulated resources. Consequently, the findings are interpreted as parent–child and life-stage differences in a Generation Z young adult and mostly Generation X parent sample, rather than as evidence of pure generational effects.

The preregistered hypotheses are:

**H1:**  *Gen X workers will have higher work satisfaction than Gen Z workers.*

**H2:**  *Core self-evaluations will be a significant predictor of work satisfaction.*

**H3:**  *Gen X workers will have higher levels of core self-evaluations than Gen Z workers.*

**H4:**  *Gen Z young adults will have lower life satisfaction than their Gen X parents.*

**H5:**  *Financial situation will predict work satisfaction.*

**H6:**  *Work satisfaction will be related to life satisfaction.*

**H7:**  *Conformity, tradition, benevolence, self-direction, and hedonism will be positively related to life satisfaction.*

**H8:**  *Achievement will be positively related to work satisfaction.*

## 2. Materials and Methods

### 2.1. Study Design, Preregistration, and Ethical Approval

This study used a preregistered, cross-sectional, observational parent–child dyadic design. The study was preregistered on OSF on 8 March 2026: https://osf.io/8dgf4. The study was approved by the University Research Ethics Committee at the University of Opole, Poland (Committee Opinion No. 161/2025, 8 January 2026). The study compares working young adults and their parents from Poland in work satisfaction, life satisfaction, core self-evaluations, life values, and financial situation.

### 2.2. Participants and Recruitment

Participants were recruited through undergraduate psychology students enrolled in the first three years of a five-year psychology programme at a university in southern Poland. Students were drawn from both full-time and weekend study programmes. Recruitment was conducted through in-class announcements by course teachers and through a faculty website bulletin board where this study was listed alongside other research opportunities conducted by university staff. Participation in this specific study was voluntary. Although students were required as part of their study programme to engage in research-related activities, they could choose among different forms of involvement, including participation in this or other studies, work as research assistants, or similar administrative research-support tasks.

Students who chose this study were asked to complete an online questionnaire and to invite one working parent to complete the corresponding parent questionnaire. Course credit for this study option was awarded only when both members of the dyad completed the questionnaires; however, students could fulfil the research-activity requirement through other available options. Parents were invited directly by the student participants, who forwarded the same study link to them. Parents received no compensation for participation. Individual survey responses were anonymised and could not be used to identify which students participated. Information needed for awarding course credit was processed separately from the study dataset after data collection had ended.

The target sample consisted of complete parent–child dyads from Poland, including working Generation Z young adults and one of their working parents. The age criterion for student participants was planned to include Generation Z respondents. Both dyad members had to be working at the time of the study, at least part-time or in limited-hours employment; temporary work and student employment were eligible. Based on the cleaned age descriptives, most parents fell within the Generation X age range. The final analytic sample consisted of 87 complete dyads.

### 2.3. Procedure

Data collection began on 13 March 2026, after preregistration. The study was presented to participants under the title “The Values in Work and Life.” Data were collected on a closed research platform of the University of Opole. The questionnaire was programmed and hosted using LimeSurvey software (Version 3.23.0+200813). Participants completed the questionnaire online; the expected completion time was approximately 15 min. The questionnaire concerned work experiences, personal values, work–life balance, work satisfaction, life satisfaction, and general well-being.

Before completing the questionnaire, participants received information about the study and provided informed consent. They were informed that participation was voluntary and that they could withdraw at any point without giving a reason. The questionnaire did not collect direct identifying information, such as names or email addresses, as part of the study responses. Participants were also informed that data would be used only for academic research purposes, stored securely, and reported only in anonymised aggregate form. No individual results were provided. Aggregate results may be made available to participants upon request by email.

Parent–child questionnaires were matched using a self-generated anonymised identification code. The code was based on stable information about the adult child and consisted of the first two letters of the child’s first name, the child’s day of birth written as two digits, the first two letters of the child’s mother’s first name, and the first two letters of the child’s city of birth. Participants were instructed to use uppercase letters only and to avoid special characters or diacritics. The same coding rule was provided to students and parents, which allowed responses to be linked within families while avoiding the collection of direct identifying information. Codes that did not follow the required procedure, including non-informative entries such as “test” or “delete this line,” were removed during data preparation.

The questionnaire included three attention-check items. Each attention check instructed participants to select a predefined response option, for example: “If you are reading this question, please select option two, ‘rather agree.’” Participants who failed more than one attention-check were excluded from the analytic dataset.

### 2.4. Measures

#### 2.4.1. Work Satisfaction

Work satisfaction was measured with a five-item scale adapted from Judge et al. [24], in the Polish-language version by Walczak [41]. Participants responded on a seven-point scale ranging from 1 (strongly disagree) to 7 (strongly agree). An example item is: “I am fairly satisfied with my current work.” The same items were administered to young adults and their parents. Two items were reverse scored. After recoding, item responses were averaged to create a composite work satisfaction score, with higher scores indicating higher work satisfaction. Internal consistency coefficient (Cronbach’s α) of the original tool was 0.88. The results for the current sample are reported in the results section.

#### 2.4.2. Life Satisfaction

Life satisfaction was assessed with the Satisfaction with Life Scale [42], using the Polish adaptation by Jankowski [43]. The scale consists of five items measuring the global cognitive evaluation of one’s life satisfaction. Participants responded on a seven-point scale ranging from 1 (strongly disagree) to 7 (strongly agree). The same items were administered to young adults and their parents.

Item responses were averaged to create a composite life satisfaction score, with higher scores indicating higher life satisfaction. The internal consistency coefficient (Cronbach’s α) of the original tool was 0.87. The results for the current sample are reported in the Results section.

#### 2.4.3. Core Self-Evaluations

Core self-evaluations were measured with the 12-item Core Self-Evaluations Scale developed by Judge et al. [22], using the Polish adaptation by Walczak and Derbis [23]. The scale assesses a broad dispositional evaluation of one’s worth, competence, control, and emotional stability. Participants responded on a five-point scale ranging from 1 (strongly disagree) to 5 (strongly agree). The same items were administered to young adults and their parents. Six items were reverse scored. After recoding, item responses were averaged to create a composite core self-evaluations score, with higher scores indicating more positive core self-evaluations. Internal consistency coefficient (Cronbach’s α) of the original tool ranged from 0.81 to 0.87. The results for the current sample are reported in the results section.

#### 2.4.4. Personal Values

Personal values were measured with the 21-item Schwartz Portrait Values Questionnaire as used in the most recent available version of the European Social Survey at the time of questionnaire preparation. The measure assesses the ten basic values from Schwartz’s value theory. In line with the preregistered hypotheses, the present study focuses on conformity, tradition, benevolence, self-direction, hedonism, and achievement.

The original ESS version uses gendered item wording, with separate male- and female-worded forms. In the present study, the items were adapted to use gender-neutral wording. For example, instead of the ESS wording “He is a person who …,” the adapted version used “This person is …”. This modification was introduced to make the items applicable to all participants while preserving the meaning of the original value portraits.

Participants rated how similar each described person was to themselves on the ESS response scale, ranging from 1 (very much like me) to 6 (not like me at all). Items were recoded so that higher scores indicated greater importance of a given value. Value scores were computed according to the ESS/Schwartz scoring approach by averaging the relevant items for each value. In line with the preregistered analysis plan, raw value scores were used in the confirmatory models. Person-mean centering was not applied because this transformation was not specified in the preregistration.

The 21-item Portrait Values Questionnaire is intentionally brief, which limits the internal consistency of several value indices. Schwartz [44] reported relatively low reliability estimates for several of the brief value indices, including self-direction (α = 0.53), conformity (α = 0.48), tradition (α = 0.37), benevolence (α = 0.61) and achievement (α = 0.52), while only the hedonism reliability was satisfactory (α = 0.79). These values indicate that the brief PVQ subscales should be interpreted as economical indicators of broad value priorities rather than highly reliable multi-item scales. Consequently, the preregistered value-related hypotheses were retained in the confirmatory analyses, but their results should be interpreted cautiously, especially for value dimensions with lower internal consistency.

#### 2.4.5. Financial Situation

Financial situation was measured by two indicators of relative earnings and one indicator of housing situation. The first relative earning item asked participants to rate their income compared with others in a similar job. The second item asked participants to rate their income compared with others with a similar level of education and work experience. Both items were answered on a five-point scale ranging from 1 (I earn much less) to 5 (I earn much more). A relative earnings score was calculated as the mean of the two items:

Relative earnings = (income compared with similar job + income compared with similar education and work experience)/2

Housing situation was assessed with one item asking participants to evaluate their current housing position and future housing prospects. Responses were coded from 1 to 4, where higher scores indicated a more secure housing situation: 1 = does not own an apartment or house and does not currently see any prospect of owning property in the future; 2 = does not own an apartment or house but expects to own property in the future; 3 = owns an apartment or house but would like to upgrade to a larger property in the future; and 4 = owns an apartment or house and does not plan to change housing situation in the future. Because the relative earnings score and the housing item used different response ranges, both components were standardized before aggregation. The financial situation composite was also calculated, as the mean of the standardized relative earnings score and the standardized housing score:

Financial situation composite = [z(relative earnings) + z(housing situation)]/2

Higher scores indicated a more favourable financial situation.

The composite was used as the preregistered financial situation variable for testing H5. The individual indicators were additionally retained for component-level inspection because they represent different reference frames: proximal occupational comparison, broader education-and-experience comparison, and housing-related security.

Demographic and occupational variables were collected to describe the sample and characterize the parent–child dyads. The role in the study was used to distinguish parents from young adults. Age was reported in years and was used to describe the generational profile of both dyad members. Gender, occupational status, and weekly working hours were used for sample description. Relationship and parental status were used to characterize participants’ family situation.

Occupational status was reported using categories distinguishing current full-time employment, current part-time employment, and other current employment, including temporary work, student work, internships, or contract-of-mandate employment. Weekly working hours were reported as a numeric estimate of average hours worked per week.

### 2.5. Sample Size and Power

The preregistered power analysis was conducted in G*Power (version 3.1.9.7) for an F test in a linear multiple regression model testing R^2^ deviation from zero. The analysis assumed a medium-to-large effect size of f^2^ = 0.25, α = 0.05, statistical power of 0.95, and five predictors. The required sample size was *N* = 85 complete dyads.

The preregistered stopping rule allowed data collection to continue until approximately 150% of the required sample size was reached. This corresponded to a maximum target of approximately 128 complete dyads. For the present Polish sample, the practical target was approximately 100–120 complete parent–child dyads, depending on recruitment feasibility and the number of dyads retained after data cleaning. The final analytic sample consisted of 87 complete dyads, exceeding the preregistered minimum of 85 dyads.

### 2.6. Statistical Analysis

All analyses were conducted in R version 4.5.0 under Windows 11 [45]. Data management was performed using the haven (version 2.5.5) and dplyr (version 1.2.1) packages. Paired robust comparisons were conducted using the WRS2 package (version 1.1.7). Linear mixed-effects models were estimated using lme4 (version 2.0-1) and lmerTest (version 3.2-1). Model estimates and confidence intervals were organized using broom.mixed (version 0.2.9.7), and model-performance indices were calculated using performance (version 0.16.0). All statistical tests were two-sided, with the significance level set at α = 0.05.

#### Data Preparation

Data preparation followed the preregistered dyadic inclusion logic. A response was treated as complete when the participant had proceeded through all questionnaire sections and had not discontinued the survey before reaching the end. Dyads were then created by matching the self-generated identification codes provided by the working student and the parent. In total, 375 questionnaire attempts were recorded. Of these, 213 responses reached the end of the questionnaire. After code-based dyad matching, 178 individual responses remained, corresponding to 89 complete parent–child dyads. This number exceeded the preregistered minimum sample size of 85 complete dyads.

Attention checks were then applied. The questionnaire included three attention-check items. Four participants failed at least one attention check, and two participants failed two attention checks. No participant failed all three attention checks. Following the exclusion rule applied to the analytic dataset, the two dyads in which one participant failed two attention checks were removed. In both cases, the participant who failed two attention checks was the parent. The final analytic sample therefore consisted of 174 individual participants nested in 87 complete parent–child dyads.

No item-level missing data were present in the analytic dataset because only participants who completed all questionnaire sections were eligible for dyad construction and analysis. Role was coded from participants’ response to the item “In which role are you participating in this study?”, with response options “Parent of a working student” and “Working student with at least one working parent.” These responses were used to distinguish the parent and young adult members of each dyad. Gender was coded from the item “Which gender do you identify with?”, with response options “Male,” “Female,” and “No answer.” Occupational variables were coded from the corresponding self-report items included in the questionnaire.

Continuous predictors were not centered or standardized before the main analyses, except where standardization was required for the construction of the financial situation composite described above. Relative earnings and housing situation were standardized before being combined into the preregistered financial situation composite. This composite was used in the confirmatory mixed-effects model testing H5. The separate financial indicators were also standardized for the component-level decomposition analysis reported after the confirmatory model. Schwartz value items were scored following the European Social Survey approach. The original response scale was recoded so that higher values indicated greater similarity to the portrait and therefore greater importance of the value. Scores for each basic value were then calculated as the mean of the relevant items. In line with the preregistered hypotheses, the analyses used the value scores for conformity, tradition, benevolence, self-direction, hedonism, and achievement. No person-mean centering of Schwartz value scores was applied. With the exception of the standardized financial situation composite and the standardized indicators used in its supplementary decomposition, continuous predictors were entered in their original scoring units. Parent was specified as the reference category for the role variable.

### 2.7. Confirmatory Analyses

The preregistered group-difference hypotheses were tested using paired Yuen’s trimmed-mean tests for dependent samples, implemented with the yuend() function from the WRS2 package. A 20% trimming proportion was specified (tr = 0.20). The analyses compared parents and young adults within the same dyad and tested differences in work satisfaction (H1), core self-evaluations (H3), and life satisfaction (H4). Two-sided tests, an α level of 0.05, and 95% confidence intervals were used. The reduced degrees of freedom relative to the original number of dyads resulted from the trimming procedure.

Effect magnitude was summarized using the explanatory measure of effect size, $\hat{\xi }$, returned by the yuend() function. This index is the square root of a robust analogue of explained variance, $\hat{\xi }=\sqrt{\hat{Var}(\hat{Y})/\hat{Var}(Y)}$, in which group locations are estimated using trimmed means and total variability using a Winsorized variance [46,47]. The index ranges from 0 to 1, with approximate values of 0.10, 0.30, and 0.50 conventionally interpreted as small, medium, and large effects, respectively. Because yuend() reports the absolute magnitude of the effect, its direction is indicated by the signed trimmed-mean difference.

Predictor hypotheses were tested using linear mixed-effects models estimated with the lmer() function from the lme4 package. Models were fitted using restricted maximum likelihood estimation with the default optimizer and convergence-control settings. A random intercept for the parent–child matching code was included to account for the non-independence of observations within dyads. The work-satisfaction model included core self-evaluations (H2), the preregistered financial situation composite (H5), achievement value (H8), and role. The life-satisfaction model included work satisfaction (H6), conformity, tradition, benevolence, self-direction, hedonism (H7), and role. Parent was used as the reference category for role.

Fixed-effect tests and denominator degrees of freedom were calculated using Satterthwaite’s approximation as implemented in lmerTest. Unstandardized regression coefficients and Wald 95% confidence intervals are reported. Model performance was summarized using marginal and conditional *R*^2^, the intraclass correlation coefficient, and root mean square error, calculated with the performance package. Marginal *R*^2^ represents the variance explained by the fixed effects, whereas conditional *R*^2^ represents the variance explained by both fixed and random effects.

The preregistered hypothesis tests were treated as confirmatory analyses. Pearson correlations and the component-level decomposition of financial situation were treated as supplementary analyses used to characterize and interpret the confirmatory findings. In the supplementary financial model, the financial situation composite was replaced by its three standardized indicators, while the other predictors and the random-intercept structure remained unchanged.

### 2.8. Use of Generative Artificial Intelligence

During preparation of the manuscript, the authors used ChatGPT (GPT-5.5 Thinking) to assist with manuscript structuring, academic language editing, and suggestions for R syntax. The tool was not used to collect data or execute statistical analyses. All AI-assisted output was reviewed, verified, and edited by the authors, who take full responsibility for the final content of the manuscript.

## 3. Results

### 3.1. Participant Flow and Exclusions

**Figure 1:**
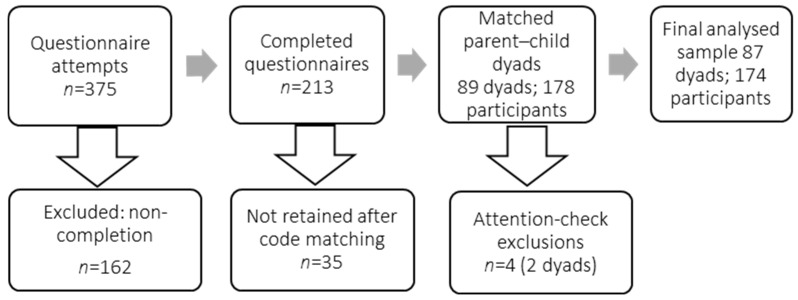
Participant flow and exclusions.

The recruited sample was substantially reduced before analysis, mainly because of incomplete questionnaires and a missing second dyad member (see Figure 1).

### 3.2. Descriptive Statistics and Reliability

The final analytic dataset, contained 87 complete parent–child dyads, corresponding to 174 individual participants. As shown in Table 1, parents were between 41 and 62 years old (*M* = 49.90, *SD* = 4.15), whereas young adults were between 18 and 30 years old (*M* = 21.41, *SD* = 2.25). Women constituted the majority of both groups. Most parents reported current full-time employment, whereas most young adults reported current part-time employment. Parents also reported more weekly working hours than young adults. The housing indicators were consistent with different life stages: most parents reported owning a flat or house and not planning to change their housing situation, whereas most young adults did not own property but expected to do so in the future.

Descriptive statistics and reliability coefficients for the main study variables are presented in Table 2. Work satisfaction, life satisfaction, and core self-evaluations showed acceptable to good internal consistency in both groups. Reliability estimates for the Schwartz value indices varied substantially and were very low for several of the brief value scores, particularly tradition in both groups and self-direction among young adults.

### 3.3. Correlations Among Main Variables

Pearson correlations among the main study variables were inspected separately for young adults and their parents. This group-specific presentation, shown in Table 3, was used because several predictors may have different theoretical relevance across dyad members, particularly financial situation.

Among young adults, life satisfaction was strongly and positively correlated with core self-evaluations, and core self-evaluations were also positively correlated with work satisfaction. Financial situation was positively correlated with both life satisfaction and core self-evaluations. The bivariate association between work satisfaction and life satisfaction was positive but did not reach conventional statistical significance. Among the preregistered value predictors, none showed a clear bivariate association with life satisfaction in the young-adults group, whereas achievement was positively correlated with hedonism but not with work satisfaction.

Among parents, life satisfaction was positively correlated with work satisfaction, core self-evaluations, and financial situation. Core self-evaluations were also positively correlated with work satisfaction and financial situation. Financial situation showed a small positive correlation with work satisfaction. Benevolence also showed a small positive correlation with work satisfaction among parents. The strongest value intercorrelations were observed between conformity and tradition, tradition and benevolence, and achievement and hedonism, consistent with the motivational proximity of several values in Schwartz’s value framework.

### 3.4. Preregistered Group Differences

Preregistered group-difference hypotheses were tested using paired Yuen’s trimmed *t*-tests, comparing parent and young adult scores within the same dyad. Positive trimmed mean differences shown in Table 4 indicate higher scores among parents than among young adults.

For life satisfaction, the trimmed mean difference between parents and young adults was small and not statistically significant, *t*(52) = 0.83, *p* = 0.412, 95% CI [−0.19, 0.47], explanatory effect size, $\hat{\xi }$ = 0.08. Thus, H4, which predicted lower life satisfaction among Gen Z young adults than among their parents, was not supported.

For core self-evaluations, parents scored significantly higher than young adults, trimmed mean difference = 0.33, *t*(52) = 4.43, *p* < 0.001, 95% CI [0.18, 0.48], explanatory effect size, $\hat{\xi }$ = 0.39. This result supported H3.

For work satisfaction, parents also scored significantly higher than young adults, trimmed mean difference = 0.47, *t*(52) = 3.03, *p* = 0.004, 95% CI [0.16, 0.78], explanatory effect size, $\hat{\xi }$ = 0.30. This result supported H1.

### 3.5. Preregistered Predictors of Work Satisfaction

A linear mixed-effects model was estimated to test the preregistered predictors of work satisfaction. Dyad was included as a random intercept. Fixed effects included core self-evaluations, the preregistered financial situation composite, achievement value, and role, as can be seen in Table 5. The model included 174 observations nested in 87 dyads.

Core self-evaluations positively predicted work satisfaction, *b* = 0.70, 95% CI [0.41, 0.99], *p* < 0.001, supporting H2. The preregistered financial situation composite did not significantly predict work satisfaction, *b* = 0.14, 95% CI [−0.10, 0.39], *p* = 0.257; therefore, H5 was not supported in the confirmatory composite model. Achievement value also did not significantly predict work satisfaction, *b* = −0.07, 95% CI [−0.21, 0.07], *p* = 0.337, so H8 was not supported. Role was not a significant predictor after controlling for the remaining variables, *b* = 0.04, 95% CI [−0.34, 0.42], *p* = 0.840. The fixed effects explained 16.6% of the variance in work satisfaction, whereas the model including the dyadic random intercept explained 32.2% of the variance. The intraclass correlation was 0.19, indicating that part of the variance in work satisfaction was attributable to differences between dyads.

To clarify the financial situation result, we conducted a component-level decomposition in which the financial situation composite was replaced by its three indicators. This analysis showed that the components were not uniform in direction. Income compared with others with a similar level of education and work experience positively predicted work satisfaction, *b* = 0.30, 95% CI [0.11, 0.49], *p* < 0.01, and housing situation also positively predicted work satisfaction, *b* = 0.29, 95% CI [0.03, 0.54], *p* < 0.05. In contrast, income compared with others in a similar job was a negative predictor, *b* = −0.29, 95% CI [−0.48, −0.11], *p* < 0.01. This decomposition suggests that the financial situation construct contains indicators with different reference frames and should not be interpreted as a single uniform material-resource effect.

### 3.6. Preregistered Predictors of Life Satisfaction

A linear mixed-effects model was estimated to test the preregistered predictors of life satisfaction. Dyad was included as a random intercept. Fixed effects included work satisfaction, the preregistered Schwartz values predicting life satisfaction—conformity, tradition, benevolence, self-direction, and hedonism—and role. The model included 174 observations nested in 87 dyads. Details can be seen in Table 6.

Work satisfaction positively predicted life satisfaction, *b* = 0.19, 95% CI [0.05, 0.33], *p* < 0.05, supporting H6. In contrast, none of the preregistered Schwartz value predictors showed a statistically significant association with life satisfaction. Conformity, *b* = 0.06, 95% CI [−0.11, 0.22], tradition, *b* = 0.00, 95% CI [−0.19, 0.19], benevolence, *b* = 0.06, 95% CI [−0.15, 0.26], self-direction, *b* = 0.11, 95% CI [−0.05, 0.28], and hedonism, *b* = 0.11, 95% CI [−0.02, 0.24], did not significantly predict life satisfaction after controlling for the remaining variables. Thus, H7 was not supported in the preregistered models using uncentered value scores.

Role was not a significant predictor of life satisfaction after controlling for work satisfaction and values, *b* = −0.17, 95% CI [−0.51, 0.17]. The fixed effects explained 7.1% of the variance in life satisfaction, whereas the conditional model, including the dyadic random effect, explained 30.0% of the variance.

### 3.7. Preregistration Compliance and Supplementary Analyses

The study was preregistered on OSF on 8 March 2026, before data collection began on 13 March 2026. The final analytic sample consisted of 87 complete parent–child dyads, exceeding the preregistered minimum sample size of 85 complete dyads. All hypothesis tests were treated as confirmatory analyses corresponding to the preregistered hypotheses. Specifically, H1, H3, and H4 were tested with paired Yuen’s trimmed *t*-tests, whereas H2, H5, H6, H7, and H8 were tested with linear mixed-effects models including dyad as a random intercept. For H5, the preregistered financial situation composite was used in the confirmatory work-satisfaction model. We additionally reported a component-level decomposition of the financial situation construct to clarify the different reference frames represented by the income and housing indicators. The descriptive statistics, correlation matrices, and component-level decomposition were used to characterize and interpret the data and were not treated as separate preregistered hypothesis tests. The statistical-model field of the preregistration contained an apparent numbering error: H4 was listed both among the paired group comparisons and among the mixed-effects models, whereas H5 was omitted from the latter list. Because H4 had already been assigned to the paired comparison and H5 explicitly specified financial situation as a predictor of work satisfaction, the second occurrence of H4 was interpreted as referring to H5.

## 4. Discussion

The present study examined differences in, and predictors of, work and life satisfaction among working young adults and their parents in a Polish sample. The findings supported four of the eight preregistered hypotheses. Parents reported higher work satisfaction and higher core self-evaluations than their young-adult children, supporting H1 and H3. Core self-evaluations were positively associated with work satisfaction, supporting H2, and work satisfaction was positively associated with life satisfaction, supporting H6. In contrast, parents and young adults did not differ significantly in life satisfaction, so H4 was not supported. The preregistered financial situation composite did not significantly predict work satisfaction, so H5 was not supported. Achievement value did not predict work satisfaction, and conformity, tradition, benevolence, self-direction, and hedonism did not significantly predict life satisfaction; therefore, H8 and H7, respectively, were not supported.

Parents reported higher work satisfaction and higher core self-evaluations than their young-adult children. The difference in work satisfaction was of approximately medium magnitude ($\hat{\xi }=0.30$), while the CSE difference was somewhat larger ($\hat{\xi }=0.39$) but remained below the conventional large-effect benchmark of 0.50. The somewhat stronger difference in CSE is theoretically important because CSE captures generalized evaluations of personal worth, competence, control, and emotional stability—resources that may shape how individuals interpret and respond to their work circumstances. Lower CSE among young adults may therefore reflect less favourable self-appraisals in domains such as perceived control, self-efficacy, emotional stability, or self-worth, which may in turn be relevant to their lower work satisfaction. However, the cross-sectional design cannot determine how or when these differences developed nor whether they reflect generational membership, age, career stage, employment stability, family role, work experience, or accumulated resources. The findings should therefore be interpreted as evidence of parent–child and life-stage differences in CSE and work satisfaction within this sample, while still highlighting CSE as a potentially important psychological factor in understanding how younger and older workers evaluate their work.

In contrast, the expected difference in life satisfaction was not supported. Young adults did not report significantly lower life satisfaction than their parents. This distinction is important because it suggests that the observed differences were more specific to work-related satisfaction and dispositional self-evaluation than to global life evaluation. One possible interpretation is that life satisfaction integrates multiple life domains and may therefore be less sensitive to work-related or role-related differences than work satisfaction. Another possibility is that young adults and parents evaluate life satisfaction against different standards, which may reduce mean-level differences despite different life circumstances. The absence of a significant difference in life satisfaction should not be interpreted as evidence that the groups were equivalent in broader mental health. The SWLS assesses a global cognitive evaluation of life and does not measure depression, anxiety, psychiatric symptoms, or functional impairment.

Although the present study assessed satisfaction-related evaluations rather than clinical mental health, the lower work satisfaction and core self-evaluations observed among young adults can be considered within a broader context of age-related psychological vulnerability. Chiappini et al. [48] describe contemporary forms of poorer mental functioning in younger populations that may be expressed through social disconnection, emotional dysregulation, diminished sense of agency, and difficulties adapting to stressful social and occupational environments. Piro et al. [49] further indicate that exposure to high-potency cannabis and novel psychoactive substances may constitute one of several contextual factors that can intensify psychological difficulties in some young people. These clinical and substance-related factors were not measured in the present study and therefore cannot be used to explain the observed parent–child differences. They nevertheless provide a broader interpretative context suggesting that lower work satisfaction and core self-evaluations among young adults may be embedded in a wider pattern of psychosocial and mental-health vulnerabilities rather than reflecting work-related processes alone.

The findings place core self-evaluations at the center of the contrast between working young adults and their parents. Parents reported higher CSE than their young-adult children, and CSE remained positively associated with work satisfaction after controlling for the preregistered financial situation composite, achievement value, and role, b = 0.70. The fixed effects explained 16.6% of the variance in work satisfaction, while the conditional R^2^ of 0.322 indicated that the fixed effects together with dyad-level differences accounted for 32.2% of the variance. The dyad ICC of 0.19 further showed that a meaningful proportion of the variance was located between parent–child pairs. These findings suggest that the lower work satisfaction observed among young adults may be related not only to age, career stage, employment stability, or family role but also to less favorable generalized evaluations of self-worth, control, competence, and emotional stability. In this sense, CSE may help to characterize an important aspect of how young adults function in work-related settings relative to their parents. At the same time, the result is only consistent with dispositional models of work satisfaction: the cross-sectional analysis cannot establish whether CSE shapes work evaluations, work experiences shape self-evaluations, or both processes operate reciprocally.

This perspective also adds to previous parent–child dyadic research. Earlier studies have mainly examined how parents’ work experiences relate to their children’s developing work orientations, adjustment, or stress. For example, Steiner et al. [12] linked parental job satisfaction with children’s work centrality, whereas French et al. [13] examined stress processes within parent–adolescent dyads. Such research highlights the family as a context in which work-related experiences are observed and interpreted. The present study addresses a somewhat different question. It did not test transmission or crossover mechanisms but showed that working young adults and their parents differed in both CSE and work satisfaction, while CSE was associated with work satisfaction across the combined sample after accounting for role and the other preregistered predictors. The dyadic design therefore helps locate these differences within matched parent–child pairs and supports the interpretation of CSE as a relevant correlate of young adults’ work-related functioning, without implying that parents’ CSE or work satisfaction directly influenced those of their children.

In the mixed-effects models, role was not significantly associated with either work satisfaction or life satisfaction after the preregistered predictors were included. This result indicates that the adjusted models did not detect an independent association between parent–child role and either outcome. However, it does not demonstrate that core self-evaluations, financial situation, work satisfaction, or values mediated or explained the observed group differences. The models were not designed as mediation analyses, and the cross-sectional data do not establish an explanatory sequence. In the work-satisfaction model, the nonsignificant adjusted role coefficient may reflect shared variance between role and the included predictors, but this possibility cannot be distinguished from limited statistical power or other aspects of model specification.

Overall, the study identified differences between parents and their young-adult children in work satisfaction and core self-evaluations, whereas no statistically significant difference in life satisfaction was detected. This pattern suggests that the parent–child contrast was more apparent in work-related evaluations and generalized self-appraisals than in global life satisfaction. However, the nonsignificant life-satisfaction result should not be interpreted as evidence that the two groups were equivalent, and the observed differences should not be attributed uniquely to generational membership. Work satisfaction was positively associated with life satisfaction, supporting H6, although the magnitude of this association was modest (*b* = 0.19). The fixed predictors explained only 7.1% of the variance in life satisfaction (*R*^2^ _marginal_ = 0.071), indicating that work satisfaction and the selected values captured only a limited part of participants’ global life evaluations. The higher conditional *R*^2^ of 0.300 suggests meaningful variation between parent–child dyads, but most individual-level variation remained unexplained. This limited explanatory power is consistent with viewing life satisfaction as a broad outcome shaped by multiple domains of functioning. In a wider clinical discussion of younger people’s mental health, Chiappini et al. [48] emphasized the relevance of social disconnection, emotional dysregulation, and stressful social and environmental conditions. Other potentially important influences not included in the present model include physical and mental health, relationship quality, family functioning, social support, work–study conflict, job insecurity, stressful life events, and substance use. Financial circumstances may also be relevant to life satisfaction, but the supplementary findings from the present study suggest that their associations are complex and may differ depending on how financial position is assessed. This complexity is considered in more detail in the following section.

The preregistered financial situation composite did not significantly predict work satisfaction; therefore, H5 was not supported. In the supplementary decomposition, however, the individual indicators showed different associations with work satisfaction. Income compared with others with a similar level of education and work experience, as well as housing situation, was positively associated with work satisfaction, whereas income compared with others in a similar job showed a negative association. These indicators appear to represent different reference frames, and the opposing directions of their coefficients may have weakened or cancelled one another when combined into a single composite. One possible interpretation is that occupation-specific income comparison and broader career-position comparison evoke different standards of adequacy or fairness. However, because the decomposition was supplementary and the confirmatory composite was nonsignificant, the component-level coefficients should be treated as hypothesis-generating rather than as established effects.

The housing result requires particular caution. In the present sample, homeownership was concentrated almost entirely among parents, whereas very few young adults owned their accommodation. Housing status was therefore closely confounded with age, family role, and life stage and cannot be treated as a directly comparable indicator of financial position across the two groups. This pattern is consistent with evidence that homeownership and housing wealth tend to accumulate across the life course and remain concentrated among older households, while younger adults often have lower accumulated wealth and face greater difficulties saving for a deposit or qualifying for mortgage finance [50]. For young adults, not owning a house or flat may therefore reflect the structural difficulty of entering the housing market rather than simply poorer financial functioning. Conversely, ownership among parents may partly represent accumulated resources and a longer period in which housing could be acquired. The positive housing coefficient may tentatively suggest that residential security is related to more favourable work evaluations, but because it emerged from a supplementary decomposition and housing status was strongly aligned with parent–child role, it should be treated as hypothesis-generating rather than as evidence of an independent effect of homeownership.

The value-based hypotheses were not supported. Achievement was not significantly associated with work satisfaction (H8), and the selected Schwartz values were not significantly associated with life satisfaction (H7). These null coefficients should not be interpreted as strong evidence that values are unrelated to satisfaction, particularly because several of the brief PVQ scales showed low internal consistency. Tradition showed particularly low internal consistency among parents (α=0.22) and young adults (α=0.33), while self-direction was also unreliable among young adults (α=0.28).

The preregistered analyses used absolute value scores, testing whether participants who endorsed a given value more strongly also reported higher satisfaction. They did not examine the relative priority of each value within an individual’s personal value hierarchy, which would require person-mean-centered scores and would constitute a different operationalization.

The present findings therefore do not determine whether satisfaction is more closely related to value realization, value congruence, or the fit between personal values and environmental opportunities. These possibilities should be examined in future studies using more reliable measures and designs explicitly developed to distinguish value importance from value enactment. Consequently, the nonsignificant value coefficients should be interpreted as inconclusive rather than as strong evidence that values are unrelated to satisfaction.

### 4.1. Practical Implications

The practical implications of the findings are most relevant for supporting young adult workers entering the labour market. The results suggest that lower work satisfaction among young adults should not be considered only as a matter of age or generational identity. Core self-evaluations were lower among young adults than among their parents and positively predicted work satisfaction across the dyadic sample. This indicates that perceived control, self-efficacy, emotional stability, and self-worth may be relevant psychological resources for young workers. Educational institutions and employers may therefore benefit from practices that strengthen early-career confidence, role clarity, feedback quality, and perceived agency at work.

The financial findings offer only limited practical guidance. Because the preregistered composite was not significant, no broad conclusion can be drawn about financial conditions as a whole. The supplementary results nevertheless suggest that pay comparisons and housing security may be relevant concerns for young adults. Organizations may therefore consider clearer pay structures as an important factor for young adults. It needs to be stated, however, that the measured exploratory associations do not demonstrate that such measures would necessarily improve work satisfaction.

#### 4.1.1. Limitations

The study has several limitations. First, its cross-sectional and observational design does not permit causal inference. The observed associations do not establish whether core self-evaluations influence work satisfaction, whether work experiences shape self-evaluations, or whether these processes operate reciprocally. The same limitation applies to the association between work satisfaction and life satisfaction.

Second, the parent–child comparison does not isolate generational membership from age, career stage, employment stability, labour-market experience, family role, or accumulated resources. The observed differences in work satisfaction and core self-evaluations should therefore be interpreted as differences between working young adults and their parents in this sample rather than as pure cohort effects. In addition, only one parent participated for each student, and the analyses were not designed to estimate actor–partner or crossover effects. The dyadic design accounts for clustering within families but does not establish interpersonal transmission between parents and children.

Third, the sample was predominantly female and was recruited through working students from a single Polish university. University-based recruitment and the availability of research credit for completing the parent–child dyad may have introduced self-selection and limit generalizability. The findings may not represent young workers who are not university students, parents outside this recruitment context, more gender-balanced populations, or families operating in other cultural and labour-market environments.

Fourth, all constructs were assessed through self-report. Although parents and young adults completed separate questionnaires, this does not eliminate response-style or common-method influences within each participant. The value results require additional caution because several brief value scales showed low reliability, particularly tradition in both groups and self-direction among young adults. Such measurement imprecision may have attenuated the estimated associations. Person-mean centering was not applied because it was not specified in the preregistration. Consequently, the analyses tested absolute value endorsement rather than the relative priority of each value within an individual’s broader value profile.

Fifth, the financial situation measure combined indicators that may reflect different constructs and comparison standards. The preregistered composite was not significantly associated with work satisfaction, whereas the supplementary component coefficients differed in direction. This pattern suggests that future studies should distinguish occupational income comparison, career-position comparison, objective income, perceived financial adequacy, and housing security rather than combining them into one broad indicator without additional validation. Housing status was also strongly confounded with age and life stage because ownership was concentrated among parents. Its association with work satisfaction cannot therefore be interpreted independently of accumulated wealth, family role, and the structural difficulties young adults may face when entering the housing market.

Sixth, the life-satisfaction model had modest explanatory power. The fixed predictors explained 7.1% of the variance, whereas the conditional R^2^ was 0.300, indicating additional between-dyad heterogeneity but leaving substantial individual-level variation unexplained. Relevant factors such as physical and mental health, relationship quality, family functioning, social support, job insecurity, work–study conflict, stressful life events, psychiatric history, substance use, and clinical symptoms were not measured. These omitted variables cannot be used to explain the present findings, but their absence limits the scope of interpretation.

Finally, the a priori power analysis was based on a conventional multiple-regression model rather than on the exact mixed-effects structure subsequently used. Although the achieved sample exceeded the preregistered minimum, the original calculation did not explicitly account for dyadic clustering, random effects, or the number of parameters in each mixed model. The study may therefore have had limited power to detect smaller fixed effects, particularly for the value predictors and the adjusted role coefficients.

#### 4.1.2. Directions for Future Research

Future research could extend the present findings in several directions. Longitudinal parent–child studies are needed to examine whether parental work satisfaction, core self-evaluations, and financial conditions predict changes in young adults’ work satisfaction and life satisfaction over time. Such designs would also allow explicit tests of intergenerational transmission models and stronger separation of age, cohort, and period effects.

Future studies would also benefit from more representative samples. The present study used a university-based recruitment route, which was useful for collecting matched dyads but limits generalizability. Representative or stratified samples could test whether the observed differences in CSE and work satisfaction are stable across educational, occupational, and socioeconomic groups. Cross-cultural comparison is another possible path, especially because the meaning of financial situation, housing prospects, and values may differ across labour-market and welfare contexts.

A further direction is a multi-measurement approach. Future studies could combine self-reports from both dyad members with behavioural, occupational, or administrative indicators, such as objective income, job contract type, housing status, or employer-reported work conditions. This would help distinguish subjective financial adequacy from objective material resources and would clarify whether satisfaction is more strongly linked to perceived conditions or to measurable work and life circumstances.

## 5. Conclusions

This preregistered parent–child study examined work satisfaction and life satisfaction among working young adults and their parents in Poland. The findings indicate that core self-evaluations were central to the observed pattern of results: parents reported higher CSE than their young-adult children, and CSE positively predicted work satisfaction across the dyadic sample. Work satisfaction, in turn, was positively related to life satisfaction, whereas the preregistered value coefficients were nonsignificant. These null findings remain inconclusive given the limited reliability of several brief PVQ scales. Overall, the study suggests that satisfaction differences in parent–child work contexts may be better understood by considering dispositional self-evaluative resources, especially CSE. Given the cross-sectional and observational design, these conclusions should be interpreted as evidence of associations rather than causal effects.

## Figures and Tables

**Table 1:** Sample Characteristics by Role.

Characteristic	Parents	Young Adults
***n***	87	87
Age, ***M*** (***SD***)	49.90 (4.15)	21.41 (2.25)
Age range	41–62	18–30
Gender: female	68 (78.2%)	73 (83.9%)
Gender: male	17 (19.5%)	10 (11.5%)
Gender: missing	2 (2.3%)	4 (4.6%)
Current full-time employment	78 (89.7%)	11 (12.6%)
Current part-time employment	6 (6.9%)	54 (62.1%)
Other current employment	3 (3.4%)	22 (25.3%)
Weekly working hours, ***M*** (***SD***)	41.67 (10.35)	27.12 (11.54)
Weekly working hours range	8–80	5–56
Relative earnings, ***M*** (***SD***)	3.01 (0.95)	2.95 (0.58)
Housing: no property, no prospect	3 (3.4%)	15 (17.2%)
Housing: no property, future prospect	8 (9.2%)	67 (77.0%)
Housing: owns property, wants upgrade	8 (9.2%)	4 (4.6%)
Housing: owns property, no planned change	68 (78.2%)	1 (1.1%)

**Table 2:** Descriptive Statistics and Reliability of Main Study Variables.

	Parents		Young Adults	
Measure	*M* (*SD*)	Cronbach’s α	*M* (*SD*)	Cronbach’s α
Work satisfaction (BJSS)	4.97 (1.10)	0.88	4.62 (1.12)	0.85
Life satisfaction (SWLS)	4.69 (1.00)	0.84	4.56 (1.15)	0.86
Core self-evaluations (CSES)	3.42 (0.55)	0.83	3.14 (0.56)	0.83
Self-direction (Schwartz)	4.40 (1.08)	0.55	4.63 (0.87)	0.28
Conformity (Schwartz)	4.00 (1.04)	0.57	3.74 (1.18)	0.64
Tradition (Schwartz)	4.08 (1.04)	0.22	3.40 (1.14)	0.33
Benevolence (Schwartz)	4.71 (0.81)	0.48	5.24 (0.77)	0.65
Hedonism (Schwartz)	3.15 (1.15)	0.75	3.83 (1.17)	0.82
Achievement (Schwartz)	3.55 (1.17)	0.74	4.59 (1.07)	0.73

**Table 3:** Pearson Correlations Among Main Variables in the group of (**a**) Parents and (**b**) Young Adults.

**(a) Pearson Correlations for Parents**									
**Variable**	**1**	**2**	**3**	**4**	**5**	**6**	**7**	**8**	**9**
1. WS	—								
2. LS	0.28 **	—							
3. CSE	0.42 ***	0.59 ***	—						
4. Fin	0.23 *	0.30 **	0.27 *	—					
5. Ach	−0.15	0.04	0.07	0.12	—				
6. Con	−0.02	0.03	−0.00	0.00	−0.05	—			
7. Trad	0.06	0.03	−0.16	0.02	−0.22 *	0.52 ***	—		
8. Ben	0.22 *	0.16	0.02	−0.12	−0.05	0.35 ***	0.43 ***	—	
9. SD	−0.03	0.09	0.25 *	−0.03	0.27 *	−0.14	−0.29 **	−0.04	—
10. Hed	−0.09	0.12	−0.03	0.11	0.39 ***	−0.15	−0.11	−0.17	0.19
**(b) Pearson Correlations for Young Adults**									
**Variable**	**1**	**2**	**3**	**4**	**5**	**6**	**7**	**8**	**9**
1. WS	—								
2. LS	0.18	—							
3. CSE	0.33 **	0.59 ***	—						
4. Fin	0.16	0.29 **	0.35 ***	—					
5. Ach	0.11	0.11	0.04	0.13	—				
6. Con	−0.06	0.05	−0.17	0.10	0.08	—			
7. Trad	0.21	0.03	−0.10	0.15	0.02	0.57 ***	—		
8. Ben	0.12	−0.02	−0.14	−0.10	−0.08	0.08	0.36 ***	—	
9. SD	0.03	0.11	0.20	−0.00	−0.07	−0.28 **	−0.32 **	−0.03	—
10. Hed	0.16	0.09	0.17	0.19	0.32 **	−0.07	0.07	−0.06	−0.11

Note. WS = work satisfaction; LS = life satisfaction; CSE = core self-evaluations; Fin = financial situation composite; Ach = achievement; Con = conformity; Trad = tradition; Ben = benevolence; SD = self-direction; Hed = hedonism. *N* = 87. * *p* < 0.05. ** *p* < 0.01. *** *p* < 0.001.

**Table 4:** Preregistered Group Differences Based on Paired Yuen’s Trimmed t-Tests.

Hypothesis (Outcome)	Trimmed Mean Diff. (Parent—Young Adult)	*t*	*df*	*p*	95% CI	Explanatory Effect Size, $\hat{\xi }$
H4 (Life satisfaction)	0.14	0.83	52	0.412	[−0.19, 0.47]	0.08
H3 (Core self-evaluations)	0.33	4.43	52	<0.001	[0.18, 0.48]	0.39
H1 (Work satisfaction)	0.47	3.03	52	0.004	[0.16, 0.78]	0.30

Note. Positive trimmed mean differences indicate higher scores among parents than among young adults. CI = confidence interval. $\hat{\xi }$ is an unsigned explanatory effect-size measure; the direction of each difference is indicated by the signed trimmed-mean difference. Approximate benchmarks of 0.10, 0.30, and 0.50 represent small, medium, and large effects, respectively.

**Table 5:** Linear mixed-effects model predicting work satisfaction.

Predictor	*b*	95% CI	Hypothesis
Intercept	2.76 ***	[1.66, 3.86]	—
Core self-evaluations	0.70 ***	[0.41, 0.99]	H2 supported
Financial situation composite	0.14	[−0.10, 0.39]	H5 not supported
Achievement	−0.07	[−0.21, 0.07]	H8 not supported
Role: young adult	0.04	[−0.34, 0.42]	Covariate

Note. The model included 174 observations nested within 87 dyads and a random intercept for the parent–child matching code. Parent was the reference category for role. The financial situation composite was calculated as the mean of standardized relative earnings and standardized housing situation. Marginal *R*^2^ *=* 0.166; conditional *R*^2^ *=* 0.322; *ICC* = 0.187; *RMSE* = 0.841. *** *p* < 0.001.

**Table 6:** Linear mixed-effects model predicting life satisfaction.

Predictor	*b*	95% CI	Hypothesis
Intercept	2.41 **	[0.95, 3.87]	-
Work satisfaction	0.19 *	[0.05, 0.33]	H6 supported
Conformity	0.06	[−0.11, 0.22]	H7 not supported
Tradition	0.00	[−0.19, 0.19]	H7 not supported
Benevolence	0.06	[−0.15, 0.26]	H7 not supported
Self-direction	0.11	[−0.05, 0.28]	H7 not supported
Hedonism	0.11	[−0.02, 0.24]	H7 not supported
Role: young adult	−0.17	[−0.51, 0.17]	Covariate

*Note.* The model included a random intercept for dyad. Marginal *R*^2^ = 0.071; conditional *R*^2^ = 0.300; *ICC* = 0.20; *RMSE* = 0.80. * *p* < 0.05. ** *p* < 0.01.

## Data Availability

The preregistration is publicly available at OSF: https://osf.io/8dgf4, accessed on 8 March 2026. The dataset analysed in the present study is not publicly available because the informed consent procedure did not include public sharing of participant-level dyadic data. Anonymised data may be made available by the corresponding author upon reasonable request, subject to ethical and privacy restrictions.

## Abbreviations

The following abbreviations are used in this manuscript:

BJSS	Brief Job Satisfaction Scale
CI	Confidence interval
CSE	Core self-evaluation
CSES	Core Self-Evaluations Scale
ESS	European Social Survey
FERS	European Funds for Social Development
Gen X	Generation X
Gen Z	Generation Z
ICC	Intraclass correlation coefficient
M	Mean
NAWA	Polish National Agency for Academic Exchange
OSF	Open Science Framework
PVQ	Portrait Values Questionnaire
R^2^	Coefficient of determination
RMSE	Root mean square error
SD	Standard deviation
SWLS	Satisfaction With Life Scale
